# Complex coronary artery disease revascularization planning with computed tomography and 3-dimensional hologram

**DOI:** 10.1016/j.xjtc.2023.04.017

**Published:** 2023-05-08

**Authors:** Tsung-Ying Tsai, Shigetaka Kageyama, Fabio Ramponi, Jagat Narula, Charles Taylor, Adam Updegrove, Scot Garg, Yoshinobu Onuma, Patrick W. Serruys, John Puskas

**Affiliations:** a Cardiovascular Center, Taichung Veterans General Hospital, Taichung, Taiwan; b CORRIB Research Centre for Advanced Imaging and Core Laboratory, University of Galway, Galway, Ireland; c Department of Cardiovascular Surgery, Mount Sinai Morningside, New York, NY; d The University of Texas Health Science Center at Houston, Houston, Tex; e HeartFlow, Inc, Mountain View, Calif; f Department of Cardiology, Royal Blackburn Hospital, Blackburn, United Kingdom; g Department of Medicine, University of Central Lancashire, Preston, United Kingdom

**Figure:**
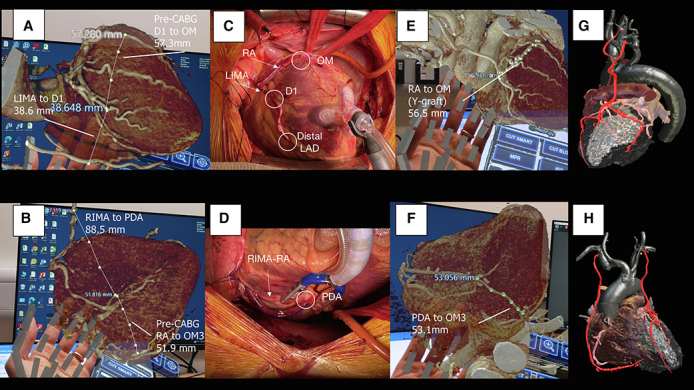
Holograms of the pre- and post-CABG CT, surgical images, and post-CABG 3D printing model.

**Central Message**

Complete revascularization of complex coronary artery disease can be planned without cine-angiography by combining CT with 3D holograms to predict the graft lengths needed for CABG.

A 71-year-old man was referred for consideration of coronary revascularization on the background of stable coronary artery disease in the context of exertional and occasional rest angina. His medical history included hypertension, dyslipidemia, well-controlled human immunodeficiency virus infection, and spinal stenosis. Per Mount Sinai Morningside Hospital regulation, additional institutional review board approval was not required. The patient provided written informed consent for publication of study data. The patient was enrolled in the FASTTRACK CABG trial (NCT 04142021), which aims to assess the feasibility of CCTA and FFR_CT_ to replace coronary angiography as CABG guidance.

A coronary computed tomography angiogram (CCTA) with CT-fractional flow reserve (FFR_CT_) was performed to plan revascularization and showed functionally significant triple-vessel coronary artery disease. There were respective fibrocalcific lesions and soft plaque in the proximal and distal parts of the right coronary artery. There were also significant fibrocalcific lesions in the left main stem, left anterior descending artery (LAD), LAD-first diagonal (D1) bifurcation, left circumflex artery, and ramus intermedius. Mixed plaques were found in the first obtuse marginal branch (OM1), with soft plaques in the distal LAD, D1, and OM1. Both anatomical and functional SYNTAX scores were 66.^1^ Thus, the heart team concluded that the patient should receive coronary artery bypass surgery (CABG). The patient was enrolled in the FASTTRACK CABG trial (NCT 04142021), which aims to assess the feasibility of CCTA and FFR_CT_ to replace coronary angiography as CABG guidance.

A 3-dimensional (3D) hologram was reconstructed from the CCTA, and preoperative measurements were performed to estimate the length of grafts needed to be harvested. The hologram reconstruction and measurements were performed using the CarnaLife Holo software (MedApp). The diameter of the right and left internal mammary arteries (RIMA, LIMA) and the distance from the internal mammary arteries to the corresponding coronary beds were measured. The distances between the LIMA to the D1 and the RIMA to the right coronary artery–posterior descending artery (PDA) were 38.6 mm and 88.5 mm, respectively. The predicted lengths for the radial artery (RA) grafts between the LAD and OM1 and the PDA and the left posterolateral artery were 57.3 mm and 51.9 mm, respectively (Figure 1, *A* and *B*; Video 1).

**Figure 1:**
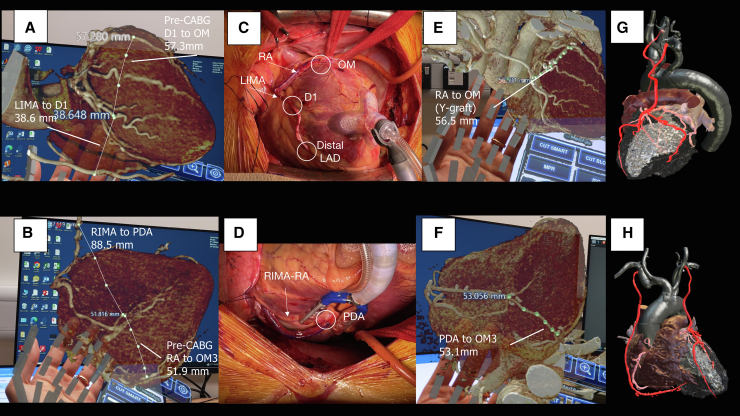
A and B, Show the 3D hologram derived from the pre-CABG CT scan with the measurements of the distance between the LIMA to D1, D1 to OM1, and RIMA to PDA to OM3 displayed. The distance between D1 to OM1 and PDA to OM3 corresponds to the length of RA grafts needed to be harvested. C and D, Show the intraoperative photographs of the LIMA, RIMA, and the 2 RA grafts. E and F, Show the lengths of the implanted RA grafts measured on the post-CABG CT hologram, which is quite similar to the pre-CABG planned lengths. G and H, Show the postprocedure 3D-reconstructed images for 3D printing preparation. *LIMA*, Left internal mammary artery; *CABG*, coronary artery bypass graft surgery; *OM1*, first obtuse marginal branch of the left circumference artery; *RIMA*, right internal mammary artery; *PDA*, posterior descending artery; *RA*, radial artery; *OM3*, third obtuse marginal branch of the left circumference artery; *LAD*, left anterior descending artery; *3D*, three-dimensional; *CT*, computed tomography; *D1*, first diagonal branch of the left anterior descending artery.

Using this information for guidance, the patient underwent 5-vessel full-arterial, no-aortic touch, off-pump CABG via a median sternotomy with the following grafts: LIMA sequentially to D1 and LAD; RA as a T graft from LIMA to OM1; and RIMA–radial extension to PDA and left posterolateral. The hologram estimation of graft lengths ensured that only the left RA needed to be harvested (Figure 1, *C* and *D*). Assessment of transit time flow showed satisfactory flow in all grafts, with low pulsatility indexes. His postoperative course was uneventful; he was extubated on day 1 postoperatively and discharged home on day 4.

At 30-day follow-up, he was angina-free, and repeated CCTA showed all grafts were patent, yielding a post-CABG SYNTAX score of 2.^1^ Another hologram was reconstructed using the post-CABG CTCA, and this showed the lengths of the RA grafts were 56.5 mm (LAD to OM1) and 53.1 mm (PDA to left posterolateral), which were similar to the pre-CABG estimations of 57.3 mm and 51.9 mm, respectively (Figure 1, *E* and *F*). A 3D-printing model was prepared for further evaluation (Figure 1, *G* and *H*).

## Discussion

This is the first case to demonstrate that planning complex coronary revascularization can be achieved purely with CCTA, FFR_CT_, and 3D holograms. CCTA has the advantage of providing simultaneously an anatomic overview and plaque composition, which are both essential for risk stratification.^2^ Moreover, CCTA enables the distal lumen of total occlusions to be visualized, which helps identify targets for CABG.^3^ FFR_CT_ provides physiological assessment on top of anatomical information. However, the complex interaction between the stenosis of the native vessel and the grafts is still difficult to predict preoperatively. According to the SYNTAX III trial, functional assessment alters the decision of the heart team in more than 7% of cases and modifies the vessel for revascularization in 12% compared with CCTA alone.^4^ The addition of 3D holograms can further empower surgeons to virtually visualize and manipulate complex anatomy by themselves before and during the actual operation.^5^ With contemporary software, they can plan the precise length of the grafts to be harvested, allowing them to make an individualized plan for their patient. This case demonstrates that visualization and measurements can be performed in real-time. It is important to emphasize that all measurements were performed with gestures and voice commands, allowing measurements without breaking sterility. This mixed-reality setting has the advantage of providing high-definition images while preserving situational awareness, which is ideal for surgeons. The current report showcases that it is safe and feasible to make complex and individualized revascularization plans without invasive coronary angiography entirely. Ultimately, it is the patient, who received expedited revascularization, was exposed to less radiation, and suffered from less tissue damage, who benefited the most from these technological advances.

## Supplementary Data

Video 1 This video shows the case information, the rationale for performing a 3-D hologram, and the final results of the analysis. Video available at: https://www.jtcvs.org/article/S2666-2507(23)00143-8/fulltext.
