# Supplementary figures and images for: Complex coronary artery disease revascularization planning with computed tomography and 3-dimensional hologram
Source: JTCVS Tech. 2023 May 8;20:96–8. doi: 10.1016/j.xjtc.2023.04.017 (PMC10405190; doi:10.1016/j.xjtc.2023.04.017)

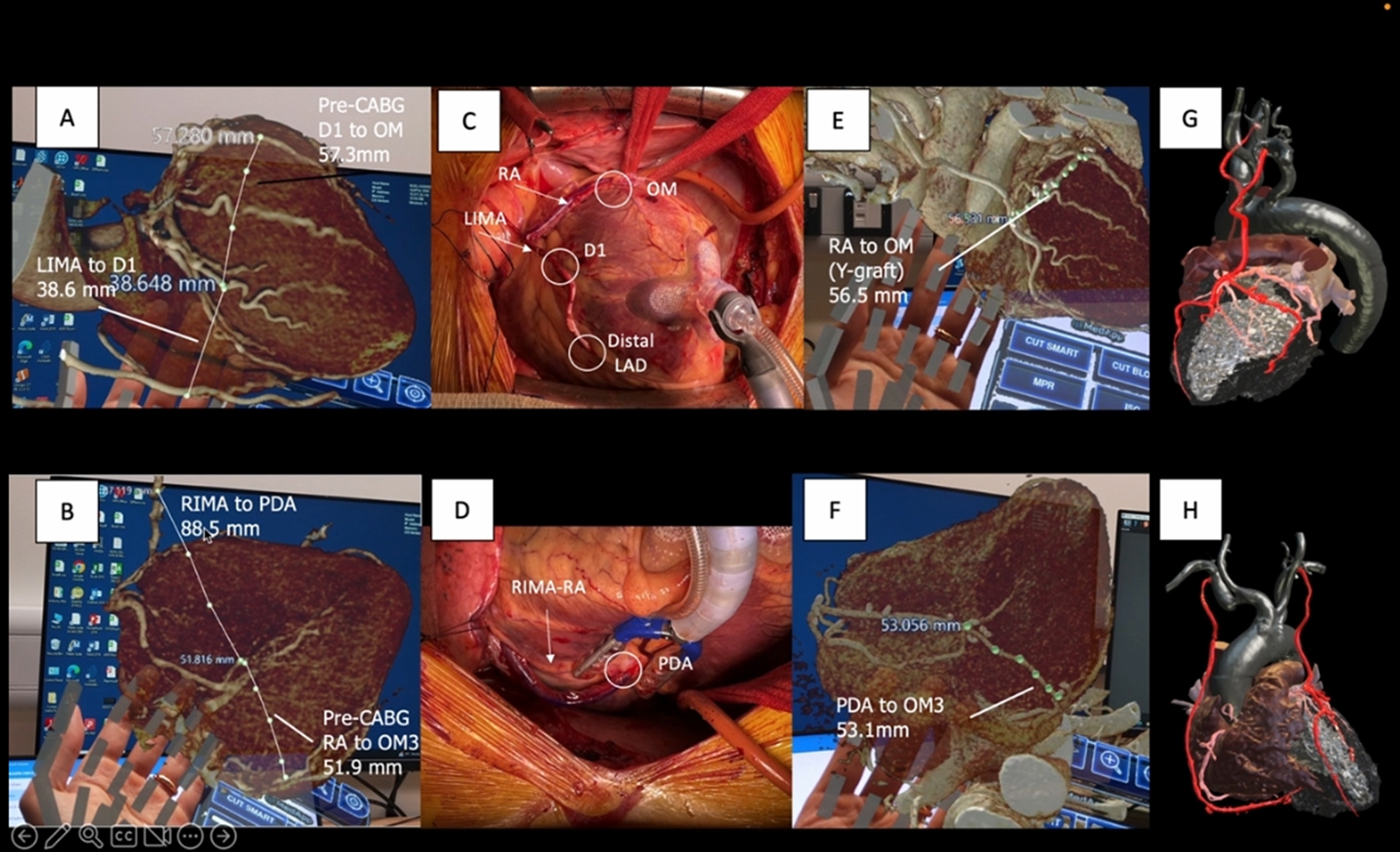

Supplement: Video 1 — This video shows the case information, the rationale for performing a 3-D hologram, and the final results of the analysis. Video available at: https://www.jtcvs.org/article/S2666-2507(23)00143-8/fulltext. [file fx2.jpg]
